# The development of a novel signature based on the m^6^A RNA methylation regulator-related ceRNA network to predict prognosis and therapy response in sarcomas

**DOI:** 10.3389/fgene.2022.894080

**Published:** 2022-10-12

**Authors:** Huling Li, Dandan Lin, Xiaoyan Wang, Zhiwei Feng, Jing Zhang, Kai Wang

**Affiliations:** ^1^ School of Public Health, Xinjiang Medical University, Urumqi, China; ^2^ School of Continuing Education, Xinjiang Medical University, Urumqi, China; ^3^ Department of Medical Engineering and Technology, Xinjiang Medical University, Urumqi, China

**Keywords:** sarcomas, m^6^A, risk score, prognosis, chemotherapy, immunotherapy

## Abstract

**Background:** N^6^ methyladenosine (m^6^A)-related noncoding RNAs (including lncRNAs and miRNAs) are closely related to the development of cancer. However, the gene signature and prognostic value of m^6^A regulators and m^6^A-associated RNAs in regulating sarcoma (SARC) development and progression remain largely unexplored. Therefore, further research is required.

**Methods:** We obtained expression data for RNA sequencing (RNA-seq) and miRNAs of SARC from The Cancer Genome Atlas (TCGA) datasets. Correlation analysis and two target gene prediction databases (miRTarBase and LncBase v.2) were used to deduce m^6^A-related miRNAs and lncRNAs, and Cytoscape software was used to construct ceRNA-regulating networks. Based on univariate Cox regression and least absolute shrinkage and selection operator (LASSO) Cox regression analyses, an m^6^A-associated RNA risk signature (m6Ascore) model was established. Prognostic differences between subgroups were explored using Kaplan–Meier (KM) analysis. Risk score-related biological phenotypes were analyzed in terms of functional enrichment, tumor immune signature, and tumor mutation signature. Finally, potential immunotherapy features and drug sensitivity predictions for this model were also discussed.

**Results:** A total of 16 miRNAs, 104 lncRNAs, and 11 mRNAs were incorporated into the ceRNA network. The risk score was obtained based on RP11-283I3.6, hsa-miR-455-3p, and CBLL1. Patients were divided into two risk groups using the risk score, with patients in the low-risk group having longer overall survival (OS) than those in the high-risk group. The receiver operating characteristic (ROC) curves indicated that risk characteristic performed well in predicting the prognosis of patients with SARC. In addition, lower m6Ascore was also positively correlated with the abundance of immune cells such as monocytes and mast cells activated, and several immune checkpoint genes were highly expressed in the low-m6Ascore group. According to our analysis, lower m6Ascore may lead to better immunotherapy response and OS outcomes. The risk signature was significantly associated with the chemosensitivity of SARC. Finally, a nomogram was constructed to predict the OS in patients with SARC. The concordance index (C-index) for the nomogram was 0.744 (95% CI: 0.707–0.784). The decision curve analysis (DCA), calibration plot, and ROC curve all showed that this nomogram had good predictive performance.

**Conclusion:** This m6Ascore risk model based on m^6^A RNA methylation regulator-related RNAs may be promising for clinical prediction of prognosis and might contain potential biomarkers for treatment response prediction for SARC patients.

## Introduction

Sarcomas (SARC) are comprised of an extensive, heterogeneous, and biologically diverse group of malignant tumors all of which are derived from mesenchymal cells (Hui, 2016). It has more than 100 different subtypes that can occur in any part of the body. Although there are more than 100 subtypes, sarcomas can be divided into two main types including soft-tissue sarcomas (STSs, accounting for 80%) and three major subtypes of bone sarcomas (BSs, including the osteosarcomas, chondrosarcomas, and Ewing’s (EW) sarcomas) (Doyle, 2014; Hui, 2016; Alavi et al., 2018; Ferguson and Turner, 2018; Hatina et al., 2019). SARC are characterized by a low incidence rate (approximately 1% of all malignancies in adults and 10–15% of all malignancies in pediatric cancers) but a poor prognosis in most cases (Hui, 2016; Siegel et al., 2019). Despite advances in the treatment of STS and osteosarcoma, with comprehensive treatment strategies including surgery, chemotherapy, radiotherapy, and targeted therapy, the survival rate of patients with advanced STSs and BSs needs to be improved (Zhu et al., 2019a; Zhang et al., 2021a; Eaton et al., 2021). Although it is difficult to determine the survival for each subtype of sarcoma due to the heterogeneity of the disease, the 5-year survival rates for patients with STS and for patients with bone sarcomas are generally larger than (>) 80% and about 70%, respectively. However, 5-year survival rates of the patients developing advanced-stage STS or various bone sarcomas are less than (<) 20% and between 22% and 57%, respectively (Bone Cancer, 2021; SarcomasTissue, 2021; SEER, 2021). In addition, about 50% of STS patients and about 90% of BS patients end up with distant metastasis, which remains a major cause of death and a barrier to effective treatment (Bacci et al., 1998; Italiano et al., 2011). Sarcomas have a poor prognosis due to their aggressive growth and high risk of metastasis (Aung et al., 2019). Therefore, further investigations into the pathogenesis of sarcomas and identification of the novel prognostic biomarkers that facilitate the improvement of therapy and prognosis of sarcomas are urgently needed.

N^6^-methyladenosine (m^6^A) is the prevalent modification in eukaryotic mRNAs, whose reversible methylation may have a profound impact on gene expression regulation (Niu et al., 2013), and plays a critical role in RNA processing. m^6^A RNA methylation is dynamically regulated by the corresponding m^6^A regulators, which are well classified into three subtypes, namely, “methylases—writer,” “demethylases—erasers,” or “m^6^A binding proteins—readers” depending on different functions (Yang et al., 2018; Chen et al., 2019a; Barbieri and Kouzarides, 2020). Increasing evidence has revealed the correlation between m^6^A and human cancers, including ovarian cancer (Li et al., 2021a), hepatocellular carcinoma (Li et al., 2021b), and colorectal cancer (Ji et al., 2020). The literature has also indicated that m^6^A-related genes might serve as novel prognostic biomarkers for different cancers, and that the m^6^A-related risk score may assist in risk assessment and prognostic stratification (Li et al., 2021c; Xu et al., 2021). The m^6^A regulators are also linked with the progression and prognosis of SARC. In recent years, there have been some studies on the role of m^6^A methylation regulators in sarcomas. For instance, WTAP, which is highly expressed in osteosarcoma tissues and associated with the worse prognosis of osteosarcoma patients, was found to potentially promote osteosarcoma progression by inhibiting HMBOX1 in an m^6^A-dependent manner *in vitro* and *in vivo* (Chen et al., 2020). Hou et al. (2020) evaluated the relationship between copy number variations (CNVs) and mutations of m^6^A regulatory factors and the prognosis of patients with soft-tissue sarcomas by using The Cancer Genome Atlas (TCGA) database. Their analysis indicated that CNVs and mutations of KIAA1429, YTHDF3, and IGF2BP1 were independent risk factors predicting OS and DFS. Zhang et al. (2021a) constructed and validated a signature based on m^6^A-related lncRNAs which could function as independent prognosis-specific predictors in STS, thereby providing novel insights into the roles of m^6^A-related lncRNAs in STS. Noncoding RNAs (ncRNAs), such as long noncoding RNAs (lncRNAs) and microRNAs (miRNAs), function as key regulators of gene expression, and the competitive endogenous RNA (ceRNA) network is a transcriptional regulatory network at the molecular level, consisting of lncRNA, miRNA, and mRNA (Salmena et al., 2011; Zhu et al., 2019b; Cava et al., 2019). More and more studies have shown that the competing endogenous RNAs (ceRNAs) have been suggested to be involved in essential biological processes and play crucial roles in the initiation and development of neoplasms, and they potentially serve as diagnostic and prognostic markers or therapeutic targets (Qi et al., 2015). At present, the mRNA–miRNA–lncRNA network is mostly studied in the ceRNA network. Recent studies have shown novel roles of the ceRNA network in thyroid cancer, colon adenocarcinoma, gastric cancer, hepatocellular carcinoma, and lung adenocarcinoma (Chang et al., 2020; Hu et al., 2020; Nie et al., 2020; Yang et al., 2020; Zhang et al., 2021b). In addition, a previous study has constructed a ceRNA network and predicted the prognosis of soft-tissue sarcoma recurrence (Huang et al., 2019). Gao et al. (2021) identified differentially expressed mRNAs (DEGs), lncRNAs (DELs), and miRNAs (DEMs) in sarcomas by comparing the gene expression profiles between sarcoma and normal muscle samples in Gene Expression Omnibus (GEO) datasets. Through target gene prediction, a lncRNA–miRNA–mRNA–ceRNA network that contained 113 mRNAs, 69 lncRNAs, and 29 miRNAs was constructed, which might provide insights into further research on the molecular mechanism and potential prognosis biomarkers. Yang et al. (2022) applied starBase and Cytoscape to construct a competing endogenous RNA (ceRNA) network based on m^6^A-related prognostic lncRNA signature and revealed the prognostic role of m^6^A-related lncRNAs in osteosarcoma and identified them as potential biomarkers for predicting the prognosis of patients with osteosarcoma. Although most studies have established prognostic models for patients with sarcoma based on the relationship between m^6^A and noncoding RNA, the biomarkers included in the models are relatively single, and the starting point and perspective of each study are different. So far as we know, the m^6^A-related ceRNA network and gene signature including three types of potential biomarkers of m^6^A regulator- and m^6^A-related noncoding RNAs (lncRNAs and miRNAs) in the regulation of soft-tissue sarcoma (STS) development and progression and prognostic values are largely unexplored. This study is based on the direction to launch a series of explorations on sarcoma.

In this study, based on transcript, somatic mutation, and clinical data obtained from The Cancer Genome Atlas (TCGA) and cBioPortal databases, we conducted extensive analysis. First, the correlation between the expression of 21 widely reported key m^6^A RNA methylation regulators and the prognosis of SARC was analyzed. Second, a regulatory network of lncRNA (predicted by the LncBase v.2 database)–miRNA (predicted by miRTarBase)–m^6^A regulators, including 16 miRNAs, 11 m^6^A regulators, and 104 lncRNAs, was constructed. In addition, using LASSO Cox regression analysis, a risk score model based on miRNA and lncRNA and m^6^A regulators in the ceRNA network was established to predict the prognosis, immune landscape, and chemosensitivity of SARC patients. Then, the risk score model was evaluated through the ROC curve. These may be potential biomarkers or therapeutic targets of SARC in the future. To further explore the potential relationship between m6Ascore and clinicopathological data, we developed a clinical m6Ascore nomogram to predict the prognosis of sarcoma patients.

## Materials and methods

### Acquisition of information of patients with SARC

**FIGURE 1:**
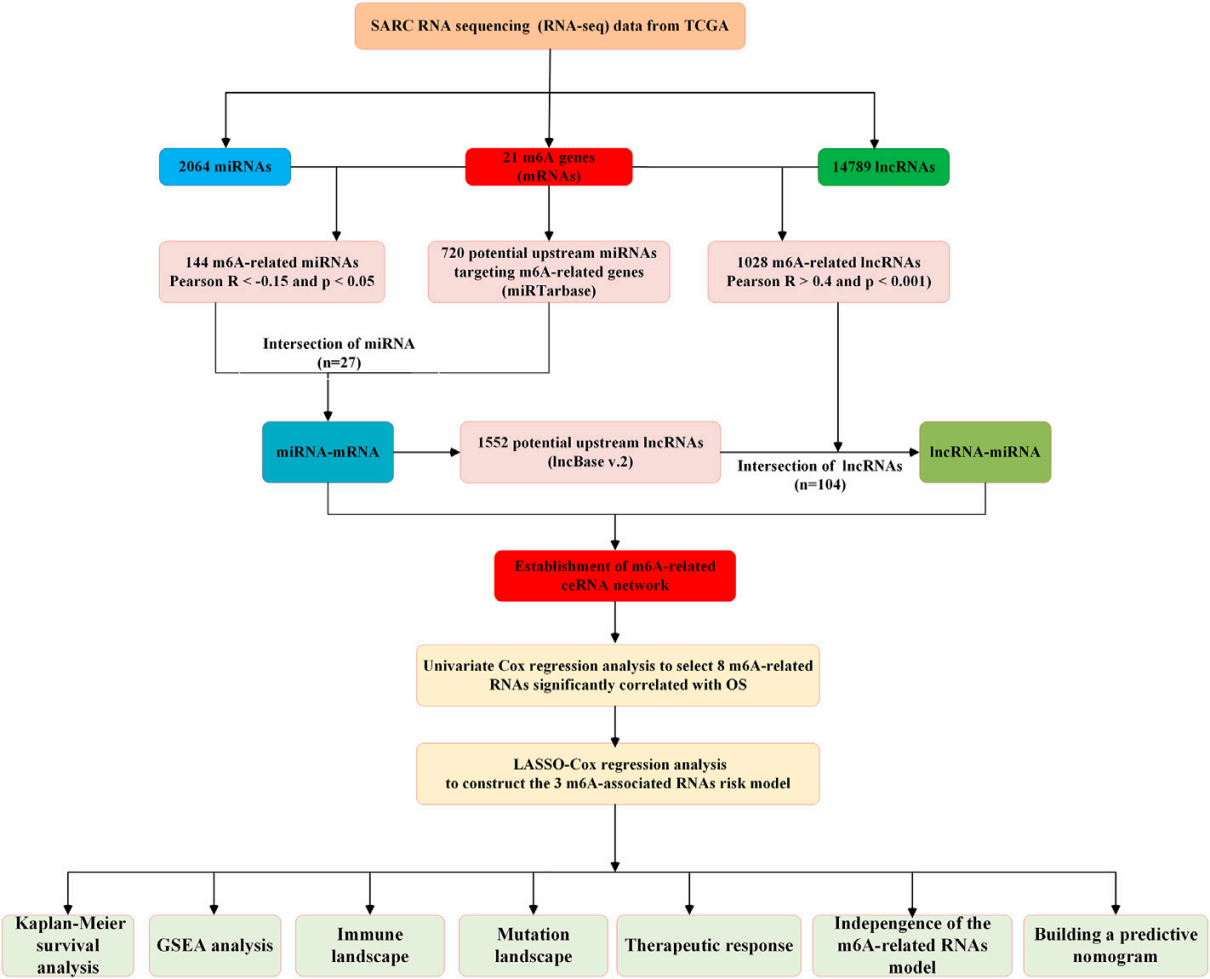
Flow chart of this study.

All datasets used in this study were publicly available, and the detailed workflow for risk model construction and subsequent analyses is shown in Figure 1. The RNA sequencing (RNA-seq) transcriptome information (including mRNA, lncRNA, and miRNA expression data), patient clinical information, and somatic mutation status data were obtained from The Cancer Genome Atlas (TCGA)(query) (https://portal.gdc.cancer.gov/) and cBioPortal websites (http://www.cbioportal.org/). Patients with unclear survival time, survival status, and clinicopathological characteristics were excluded. Only patients with overall survival (OS) times of more than or equal to 30 days were included in the dataset.

### Selection of m^6^A genes and m^6^A-related lncRNAs and miRNAs

According to other published studies, 21 m^6^A genes were investigated in this study, including writers (*METTL3*, *METTL14*, *CBLL1*, *VIRMA [KIAA1429]*, *RBM15*, *RBM15B*, *ZC3H13*, and *WTAP*), readers (*YTHDC1*, *YTHDC2*, *YTHDF1*, *YTHDF2*, *YTHDF3*, *IGF2BP1*, *HNRNPA2B1*, *HNRNPC*, *FMR1*, *LRPPRC*, and *ELAVL1*), and erasers (*ALKBH5* and *FTO*). Using the STRING (version 11.0, http://string-db.org/) database, we retrieved the interactions of the 21 m^6^A RNA methylation regulators and visualized the interactions. The Pearson correlation analysis was used to elucidate the correlation between different m^6^A RNA methylation regulators. We downloaded the profiles of lncRNAs, miRNA, and m^6^A genes from TCGA database. Thereafter, the Pearson correlation analysis was conducted to screen the m^6^A gene-related lncRNAs and miRNAs in SARC samples. LncRNAs with correlation coefficients >0.4 and *p* < 0.001 were regarded as m^6^A-related lncRNAs, while miRNAs with correlation coefficients <–0.15 and *p* < 0.05 were regarded as m^6^A-related miRNAs.

### Construction of the m^6^A-associated ceRNA network

All miRNAs that were negatively correlated with the 21 selected m^6^A regulators (*METTL3*, *METTL14*, *CBLL1*, V*IRMA [KIAA1429]*, *RBM15*, *RBM15B*, *ZC3H13*, *WTAP*, *YTHDC1*, *YTHDC2*, *YTHDF1*, *YTHDF2*, *YTHDF3*, *IGF2BP1*, *HNRNPA2B1*, *HNRNPC*, *FMR1*, *LRPPRC*, *ELAVL1*, *ALKBH5*, and *FTO*) were obtained from TCGA-SARC dataset (Pearson correlation coefficient < –0.15 and *p* < 0.05). Then, the related miRNAs possessing potential binding sites with the 21 selected m^6^A regulators were simultaneously predicted using the miRTarBase database (http://mirtarbase.cuhk.edu.cn/), and the intersection with the results from the analysis in the previous step was collected. Meanwhile, all lncRNAs were obtained from TCGA-SARC dataset and further screened according to their Pearson correlation coefficients (Pearson correlation coefficient >0.4, *p* < 0.05) with 21 selected m^6^A regulators. Next, the related lncRNAs possessing potential binding sites with the interactive m^6^A-related miRNAs were predicted using the LncBase v.2’s experimental module (http://carolina.imis.athena-innovation.gr/), and the intersection with the results from the analysis in the previous step was obtained. Finally, the lncRNA–miRNA–m^6^A regulator ceRNA network based on the foundation of the interactions between m^6^A-related lncRNA and m^6^A-related miRNA and between m^6^A-related miRNA and m^6^A regulators was created and visualized using the “ggalluvial” R package.

### Establishment of the m^6^A-associated risk model

First, by means of the “survival” package in R, univariate Cox regression analysis was utilized to explore the correlation between the genes in the ceRNA network and overall survival (OS) of SARC patients to determine the prognostic-related biomarkers. Then, we used the “glmnet” R package to perform LASSO regression analysis on the genes screened in the univariate Cox regression analysis. Finally, the genes that can be used as independent prognostic factors of OS were screened using multivariate Cox regression analysis, and their regression coefficients (β) were calculated. The following formula was used to calculate the prognostic risk score of each patient:

Risk score = β (mRNA1) * expr (mRNA1) + β (mRNA2) * expr (mRNA2) + … + β (mRNAn) * expr (mRNAn) + β (miRNA1) * expr (miRNA1) + β (miRNA2) * expr (miRNA2) + … + β (miRNAn) * expr (miRNAn) + β (lncRNA1) * expr (lncRNA1) + β (lncRNA2) * expr (lncRNA2) + … + β (lncRNAn) * expr (lncRNAn).

At the same time, the cut-off point of risk score was picked using the “survminer” R package which divided patients into high- and low-risk groups. Subsequently, the Kaplan–Meier survival analysis and log-rank test were used to evaluate the difference in the overall survival (OS) between the risk score groups. Finally, to reflect the prediction ability of the risk score model, we generated the area under the curve (AUC) of the time-dependent receiver operating characteristic (tROC) curves (“riskRegression” package in R) and calculated the area under the curve (AUC) for 1-year, 3-year, and 5-year overall survival (OS).

### Analysis of tumor immune signatures and function enrichment for m6Ascore

Tumor immune signatures were evaluated in two aspects: 1) immune checkpoints and 2) the levels of infiltrating immune and immune and stromal scores were calculated using CIBERSORT, TIMER, ssGSEA, and ESTIMATE algorithms. Parts of the results are available at the Genomic Data Commons (GDC, https://gdc.cancer.gov/) and Tumor IMmune Estimation Resource (TIMER2.0, http://timer.cistrome.org/). The gene set enrichment analysis (GSEA) was utilized to understand the biological processes involved in the high- and low-risk groups. Hallmarks in GSEA were used to identify predefined gene sets. A pathway with a |normalized enrichment score (NES)| >1.5, a *p*-value < 0.05, and a false discovery rate (FDR) < 0.05 was considered to be significant, as described in the *Results* section.

### Analysis of the tumor mutation status in the low- and high-risk groups

The information of somatic mutations in TCGA samples was downloaded from cBioPortal database and TCGA database. The tumor mutational burden (TMB) is defined as the total number of gene mutations per million bases, including the total number of gene-coding errors, base substitutions, and gene insertions or deletions. The TMB value of each patient was calculated using the Perl programming language. Significantly mutated genes between the low- and high-m6Ascore groups and the interaction effect of gene mutations were analyzed using “maftools” R packages. The statistical test for the proportion of mutation was evaluated by the two-side Chi-squared test, and *p* < 0.05 was considered to be significant.

### Prediction of therapeutic sensitivity in patients with different m6Ascore

We studied the predictive capacity of m6Ascore in responding immunotherapy and 138 drugs of chemotherapies/targeted therapies. Based on the public pharmacogenomics database, Genomics of Drug Sensitivity in Cancer (GDSC, https://www.cancerrxgene.org), the 50% inhibiting concentration (IC_50_) values of the 138 drugs were calculated using the “pRRophetic” R packages. The potential response of patients to immunotherapy was inferred by the tumor immune dysfunction and exclusion (TIDE) algorithm. Generally, a lower TIDE score predicts a better response to immunotherapy. The results of TIDE module analysis of patients with SARC from TCGA dataset were downloaded from the TIDE website (http://tide.dfci.harvard.edu/).

### Construction of a predictive nomogram

First, we developed univariate and multivariate Cox regression analyses of the m6Ascore risk signature and other clinicopathological characteristics to confirm the independence of the m6Ascore risk signature. Then, we used the aforementioned factors to establish a nomogram using the “rms” and “regplot” R packages to predict the prognosis of patients with sarcoma. Finally, ROC, C-index, calibration curve analysis, and DCA curves were used to determine whether our established nomogram was suitable for clinical use.

### Statistical analysis

The continuous data are expressed as the mean (standard deviation, SD). The categorical data are expressed as frequency and percentage. The relativity between m6Ascore risk signature and immune checkpoint molecules, and TMB were analyzed using the Spearman or Pearson correlation analysis. The Chi-squared test was used to compare different proportions. The differences in proportions of the immune-infiltrating cells, immune checkpoint gene expression, TMB, IC_50_, and TIDE-related signatures between high- and low-risk groups were compared using the Wilcoxon rank-sum test (Mann–Whitney U test). The Kaplan–Meier survival analysis and log-rank tests were used to analyze the differences in OS between different risk score groups. The univariate and multivariate Cox regression analyses were performed to screen the independent predictors for OS. In all statistical results, except for the special instructions, a two-tailed *p*-value less than 0.05 indicated statistical significance. All analyses were performed using R software (version 3.6.2, https://www.r-project.org/).

## Results

### Prognostic analysis of m^6^A-associated genes in SARC

**FIGURE 2:**
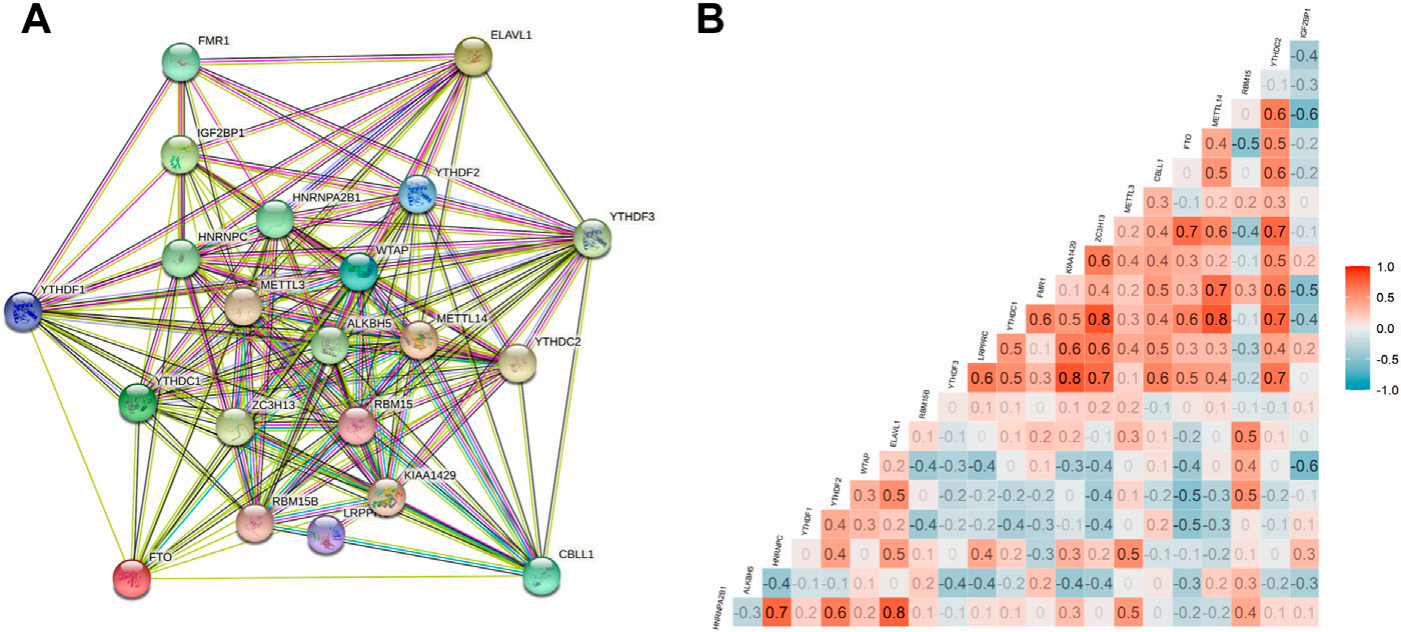
Interactions and correlations among m^6^A RNA methylation regulators in SARC. **(A)** PPI network was constructed to evaluate the interactions among m^6^A RNA methylation regulators; and **(B)** Pearson correlation analysis was used to analyze the correlations among m^6^A RNA methylation regulators.

**FIGURE 3:**
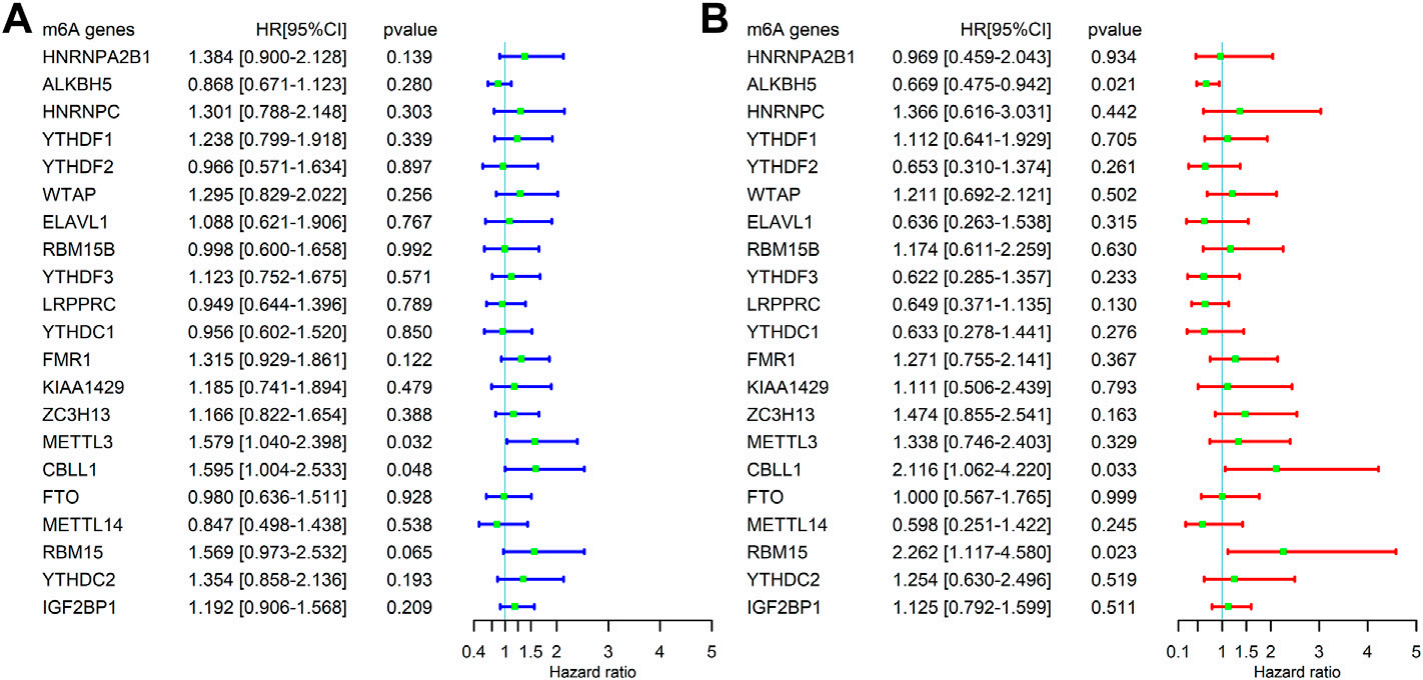
Correlation between the expression levels of m^6^A-related genes and overall survival (OS) rates in SARC patients (*N* = 245). **(A)** Univariate analysis of m^6^A-related genes associated with OS; **(B)** and multivariate analysis of m^6^A-related genes associated with OS.

A total of 255 patients with primary sarcoma had completed clinical data, including 117 (45.9%) males and 138 (54.1%) females, with the mean age being 60.7 ± 14.7. The detailed demographic and clinicopathological data of these SARC patients are shown in Table 1. With the STRING database used to understand the interaction between the 21 m^6^A RNA methylation regulators, a PPI network was obtained (Figure 2A), which indicated that all enrolled 21 studied genes exhibited gene–gene interactions. In addition, we examined the relationship between 21 regulators through the Pearson correlation test and observed that all m^6^A RNA methylation regulators were generally positively correlated (Figure 2B). *YTHDC1* showed a positive correlation with *ZC3H13* (correlation coefficient: 0.8) and *METTL14* (correlation coefficient: 0.8). *YTHDF3* showed a positive correlation with *KIAA1429* (correlation coefficient: 0.8). *HNRNPA2B1* showed a positive correlation with *ELAVL1* (correlation coefficient: 0.8). Next, according to the aforementioned criteria, we analyzed the prognostic role of the 21 m^6^A-associated genes in SARC in a total of 245 patients with OS and RNA-seq data. We performed univariate and multivariate Cox regression analyses to explore whether these genes were associated with the prognosis of SARC patients. The results of univariate Cox regression revealed that *METTL3* (*p* = 0.032) and *CBLL1* (*p* = 0.048) were risk genes for SARC (Figure 3A). The multivariate Cox regression results revealed that both *CBLL1* (*p* = 0.033) and *RBM15* (*p* = 0.023) were risk factors for OS. It also revealed that *ALKBH5* (HR (hazard ratio) = 0.669, *p* = 0.021, and 95% CI (confidence interval) [0.475–0.942]) may be a protective gene for OS (Figure 3B).

**TABLE 1:** Main clinicopathological characteristics of the 255 SARC patients.

Characteristic	Number of patients (*N* = 255)
Age	
Mean (SD)	60.7 (14.7)
Sex, n (%)	
Female	138 (54.1%)
Male	117 (45.9%)
Race, n (%)	
Asian	6 (2.4%)
Black or African American	18 (7.1%)
White	223 (87.5%)
Unknown	8 (3.1%)
Cancer status, n (%)	
Tumor free	128 (50.2%)
With tumor	98 (38.4%)
Unknown	29 (11.4%)
Radiation therapy, n (%)	
Yes	68 (26.7%)
No	176 (69.0%)
Unknown	11 (4.3%)
Cancer type, n (%)	
Dedifferentiated liposarcoma	59 (23.1%)
Desmoid/aggressive fibromatosis	2 (0.8%)
Leiomyosarcoma	100 (39.2%)
Malignant peripheral nerve sheath tumor	9 (3.5%)
Myxofibrosarcoma	25 (9.8%)
Synovial sarcoma	10 (3.9%)
Undifferentiated pleomorphic sarcoma/malignant fibrous histiocytoma/high-grade spindle cell sarcoma	50 (19.6%)

### Identification of key upstream miRNAs of 21 m^6^A genes

**FIGURE 4:**
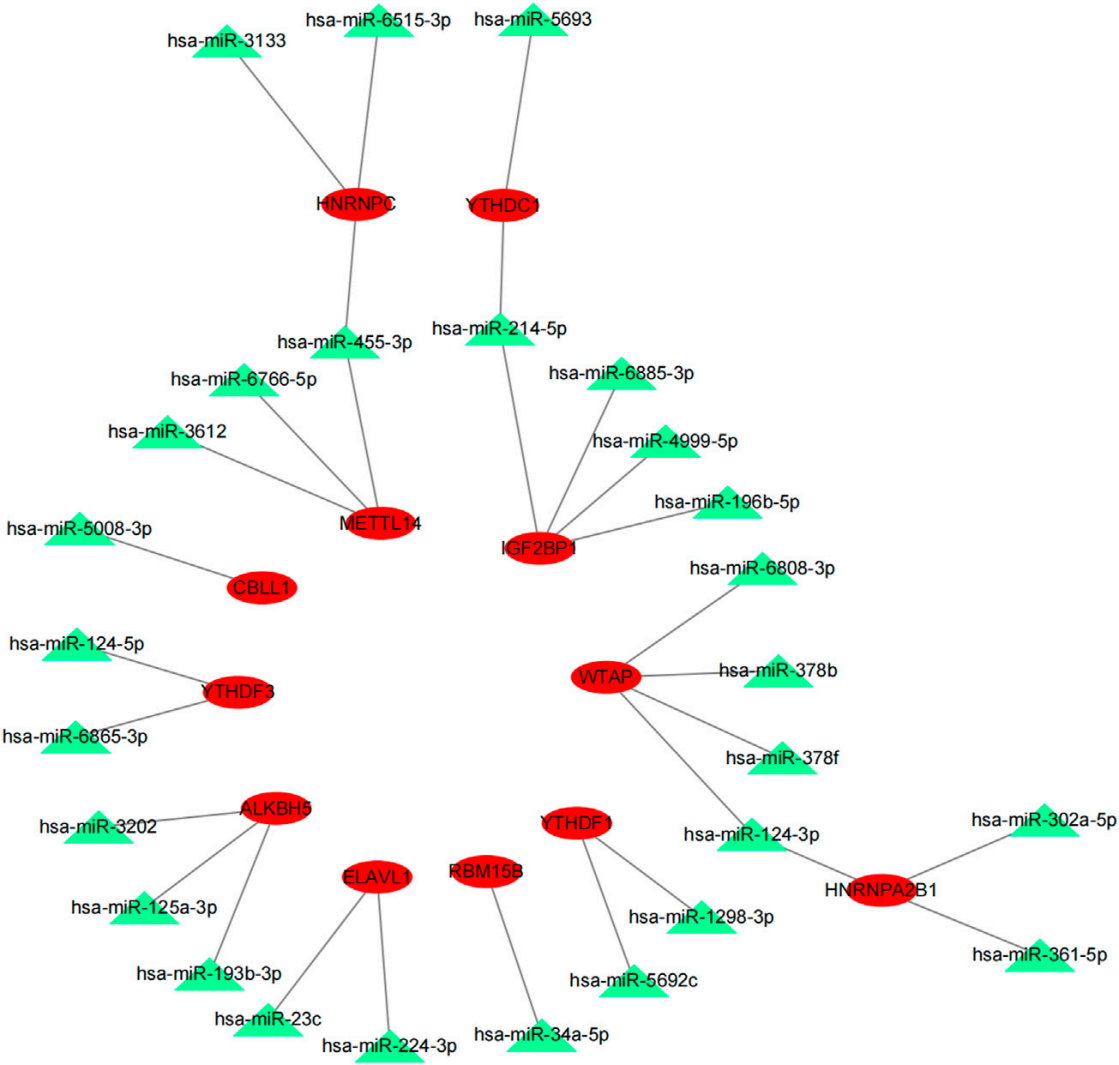
MiRNA–mRNA interaction network of m^6^A genes.

The potential upstream miRNAs of 21 m^6^A genes were predicted using the experimentally verified microRNA–target gene interaction database miRTarBase. Ultimately, it found that 720 miRNAs interacted with 20 m^6^A genes (writers: *METTL3*, *METTL14*, *CBLL1*, *KIAA1429 [VIRMA]*, *RBM15B*, *ZC3H13*, and *WTAP*; readers: *YTHDC1*, *YTHDC2*, *YTHDF1*, *YTHDF2*, *YTHDF3*, *IGF2BP1*, *HNRNPA2B1*, *HNRNPC*, *FMR1*, *LRPPRC*, and *ELAVL*; and erasers: *ALKBH5* and *FTO*), while no upstream miRNA interacted with one m^6^A gene (RBM15) in the database (Supplementary Table S1). Next, the matrix expressions of 21 m^6^A genes and 2,064 miRNAs were abstracted from TCGA database. We defined the miRNAs that were significantly related to one of the 21 m^6^A genes (Pearson R < -0.15 and *p* < 0.05) as m^6^A-related miRNAs. Finally, 144 miRNAs were discerned as m^6^A-related miRNAs (Supplementary Table S2). Also, the predicted miRNAs were then intersected with these m^6^A-related miRNAs to select the 27 m^6^A-related miRNAs (key miRNAs) that interacted with 12 m^6^A genes. Cytoscape was used to construct an miRNA–mRNA regulatory network, including 30 miRNA–mRNA pairs (Figure 4).

### Identification of upstream lncRNAs of key miRNAs in patients with SARC

**FIGURE 5:**
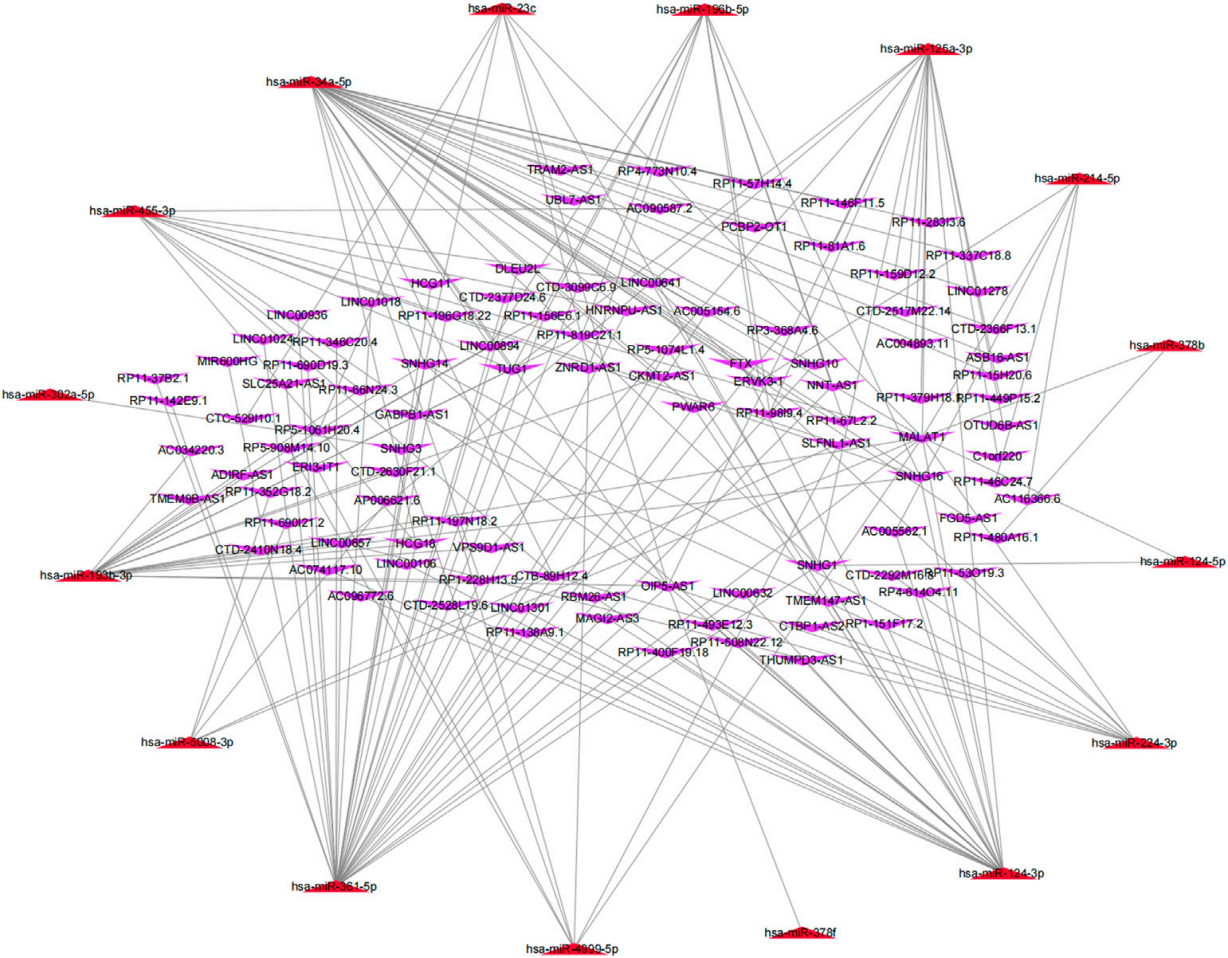
MiRNA–lncRNA regulatory network associated with m^6^A genes in SARC.

Based on the ceRNA hypothesis that lncRNAs can compete with mRNAs for miRNA binding, thus playing a vital role in the process of tumor development and progression (Nie et al., 2020), this study further used the experimental module of LncBase database (http://carolina.imis.athena-innovation.gr/diana_tools/web/index.php?r *= Lncbasev2%2Findex-experimental*), an online database, to predict the upstream lncRNAs that may interact with the 27 key miRNAs (hsa-miR-124-3p, hsa-miR-455-3p, hsa-miR-34a-5p, hsa-miR-214-5p, hsa-miR-124-5p, hsa-miR-302a-5p, hsa-miR-3202, hsa-miR-125a-3p, hsa-miR-224-3p, hsa-miR-4999-5p, hsa-miR-6766-5p, hsa-miR-6808-3p, hsa-miR-3133, hsa-miR-6885-3p, hsa-miR-1298-3p, hsa-miR-5692c, hsa-miR-378f, hsa-miR-378b, hsa-miR-5693, hsa-miR-3612, hsa-miR-6515-3p, hsa-miR-361-5p, hsa-miR-5008-3p, hsa-miR-6865-3p, hsa-miR-193b-3p, hsa-miR-23c, and hsa-miR-196b-5p), and a total of 1,552 lncRNAs were obtained (Supplementary Table S3). Then, the matrix expressions of 21 m^6^A genes and 14,789 lncRNAs were abstracted from TCGA database. We defined the lncRNAs that were significantly related to one of the 21 m^6^A genes (Pearson R > 0.4 and *p* < 0.001) as m^6^A-related lncRNAs. Finally, 1,028 lncRNAs were discerned as the m^6^A-related lncRNAs (Supplementary Table S4). Also, the predicted lncRNAs were then intersected with these m^6^A-related lncRNAs to select the m^6^A-related lncRNAs (n = 104) that interacted with 16 m^6^A-related miRNAs. Cytoscape was used to construct an lncRNA–miRNA regulatory network, including 189 lncRNA–miRNA pairs (Figure 5).

### Construction of an m^6^A-related competing endogenous RNA (ceRNA) network

**FIGURE 6:**
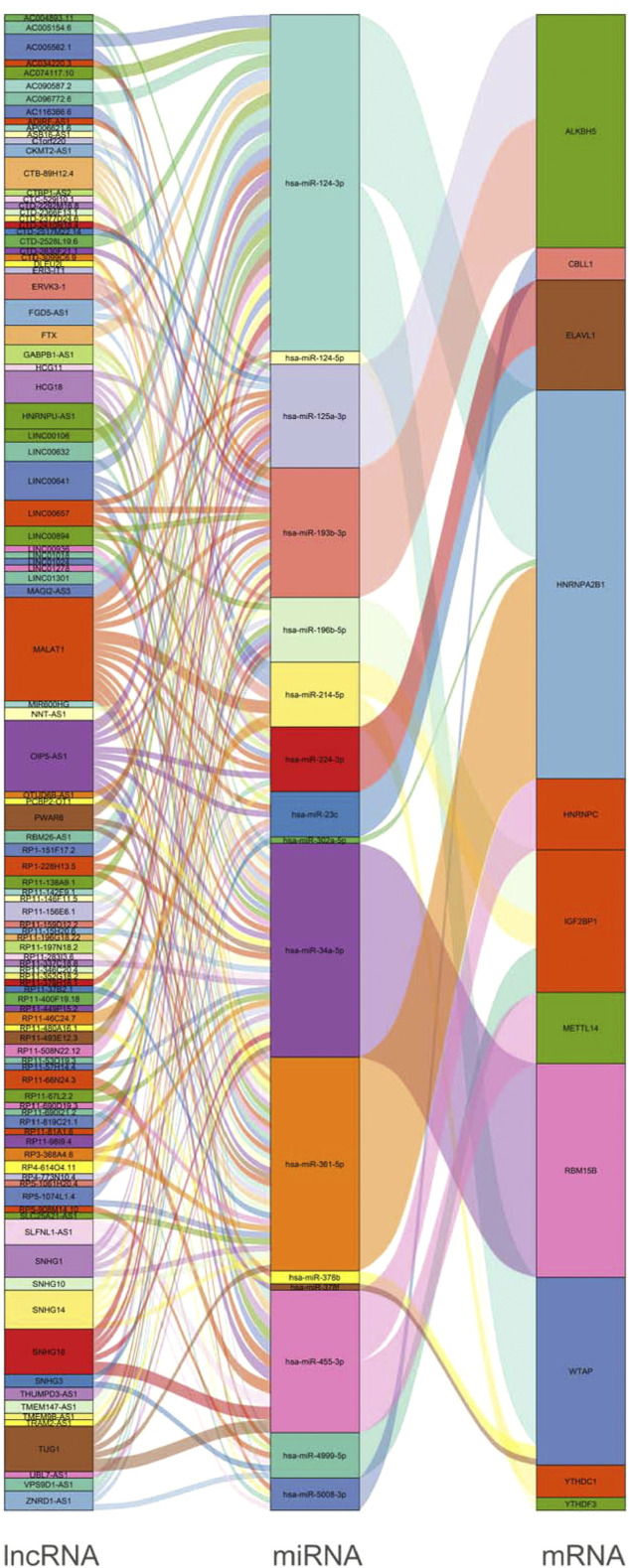
Construction of a network of lncRNA–miRNA–m^6^A regulators.

Based on the results mentioned previously, the miRNA–mRNA and lncRNA–miRNA were selected to establish an m^6^A-associated ceRNA network that contained 104 lncRNAs, 16 miRNAs, and 11 mRNAs (Figure 6). We searched the lncRNAs and miRNAs in our study in the m6A-Atlas database (Tang et al., 2021), RMBase v2.0 (Xuan et al., 2018) and WHISTLE (Chen et al., 2019b) database, which were used to predict m^6^A methylation sites in these RNAs. The search results (Supplementary Tables S6, S7) showed that most lncRNAs in this study had potential methylation sites. However, we did not retrieve the potential m^6^A methylation sites of miRNAs in these libraries. Detailed information of the ceRNA network is listed in Supplementary Table S5.

### Construction of a risk model according to m^6^A-related RNAs in SARC patients

**FIGURE 7:**
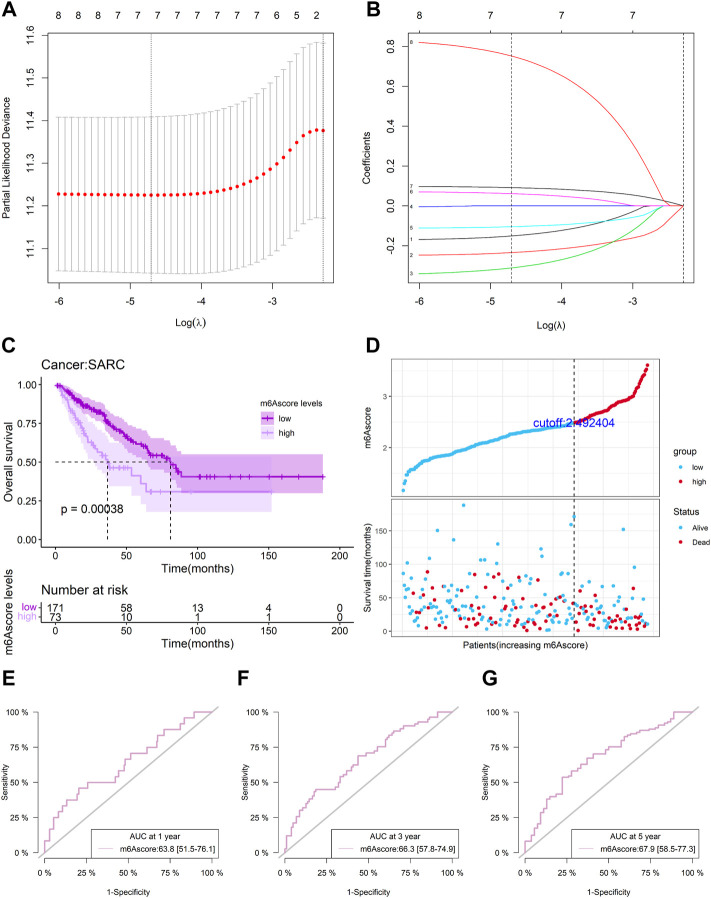
Risk model from m^6^A-related genes. **(A-B)** LASSO Cox regression analysis of eight m^6^A-related genes; **(C)** overall survival analysis for patients in high/low-risk groups; **(D)** distributions of risk score, different patterns of survival status, and survival time between the high- and low-risk groups; **(E-G)** and time-dependent ROC curves at 1, 3, and 5 years based on the m^6^A-related gene signature.

A total of 244 patient samples were included in the whole dataset. A total of 16 miRNAs, 104 lncRNAs, and 11 m^6^A regulators (mRNAs) in the ceRNA network were selected as candidate biomarkers for the following step analysis. The result of univariate survival analysis showed that five m^6^A-related lncRNAs (RP11-46C24.7, RP11-283I3.6, SLC25A21-AS1, RP11-81A1.6, and RP11-346C20.4), two m^6^A-related miRNA (hsa-miR-455-3p and hsa-miR-124-3p), and one m^6^A regulator (CBLL1) were associated with SARC prognosis (*p* < 0.1). Among these biomarkers, hsa-miR-455-3p (miRNA), hsa-miR-124-3p (miRNA), and CBLL1 (mRNA) were considered as risk biomarkers (HR > 1). Subsequently, to obtain the most useful predictive features, LASSO Cox regression analysis was performed on eight genes (Figures 7A,B). Based on the final Cox regression model results (Table 2), we selected 3 genes (*hsa-miR-455-3p*, *CBLL1*, and *RP11-283I3.6*) with *p* < 0.1 to construct the risk score model. Furthermore, we calculated the risk score (m6Ascore) of each sample based on the risk score model, and the formula was shown as follows:

**TABLE 2:** Cox proportional hazard regression analysis and LASSO of eight genes.

*Gene*	Univariate Cox regression analysis			LASSO coefficient	Multivariate Cox regression analysis		
	HR	95% CI	P		HR	95% CI	P
hsa-miR-455-3p	1.13	1.03–1.23	0.013	0.09173461	1.11	1.00–1.22	0.040
RP11-46C24.7	0.64	0.45–0.91	0.014	−0.23437212	0.78	0.50–1.20	0.251
RP11-283I3.6	0.67	0.45–0.98	0.039	−0.31135641	0.70	0.47–1.06	0.091
SLC25A21-AS1	0.74	0.55–0.99	0.040	−0.10453847	0.89	0.60–1.33	0.575
CBLL1	1.60	1.00–2.54	0.048	0.75233906	2.33	1.43–3.81	0.001
RP11-81A1.6	0.75	0.56–1.01	0.057	−0.15084732	0.84	0.56–1.25	0.388
RP11-346C20.4	0.74	0.54–1.02	0.066	—			
hsa-miR-124-3p	1.21	0.98–1.50	0.075	0.06160625	1.08	0.86–1.36	0.531

HR, hazard ratio; CI, confidence interval.

Risk score = (0.09964) * hsa-miR-455-3p + (0.84723) * CBLL1 + (−0.35103) * RP11-283I3.6.

Patients with SARC were divided into the low-risk or high-risk groups with the optimal cutoff (2.492404) of the risk score. The Kaplan–Meier (KM) curve analysis result showed that the low-risk group had a better prognosis than the high-risk group (*p* = 0.00038) (Figure 7C). The distribution of risk score between low-risk and high-risk groups is depicted in Figure 7D, and a survival status map was plotted to demonstrate the status for each sample using TCGA sarcoma dataset values. A time-dependent ROC curve was performed to evaluate the sensitivity and specificity of the risk score. The m^6^A-related signature’s AUC values were 0.638 (0.515, 0.761), 0.663 (0.578, 0.749), and 0.679 (0.585, 0.773), respectively, for an OS of 1, 3, and 5 years (Figures 7E–G). This indicates that our risk score model could be used to predict SARC patient survival.

### m6Ascore was associated with the SARC immune landscape

**FIGURE 8:**
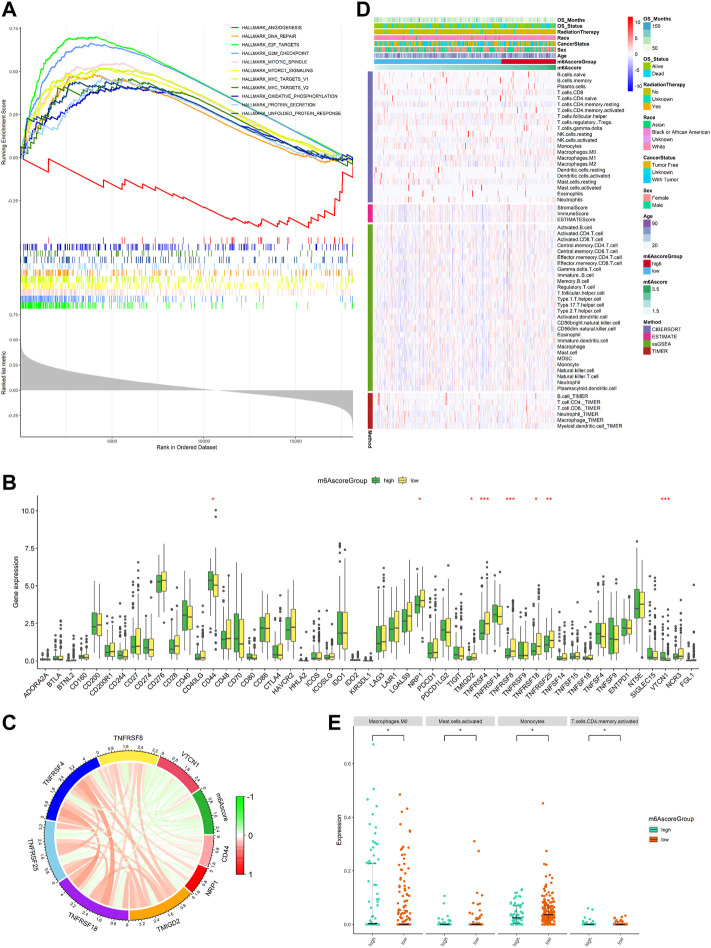
Function enrichment analysis for m6Ascore and immune landscape of different m6Ascore subgroups. **(A)** GSEA of samples with high or low m6Ascore. Top ten significant pathways associated with high m6Ascore (*p* < 0.05 and FDR-adjusted q < 0.05, NES > 1.5). Significant pathways in the red module are associated with low m6Ascore (nominal *p* < 0.05 and FDR adjusted q < 0.05, NES < 1.5); **(B)** comparison of immune checkpoint gene expression levels between the low-m6Ascore group and the high-m6Ascore group. *p < 0.05; **p < 0.01; ***p < 0.001; **(C)** correlation chord chart shows the mutual correlation between m6Ascore and several prominent immune-checkpoint-relevant genes (*CD44*, *VTCN1*, *TMIGD2*, *TNFRSF18*, *TNFRSF25*, *TNFRSF4*, *TNFRSF8*, and *VTCN1*); **(D)** heatmap for immune responses based on CIBERSORT, ESTIMATE, ssGSEA, and TIMER algorithms among the high- and low-risk group; and **(E)** beeswarm plot for infiltration levels of immune cells in high- and low-risk samples through the CIBERSORT. The statistical difference between the two groups was compared by the Wilcoxon rank-sum test. *p < 0.05; **p < 0.01; ***p < 0.001.

Our study explored the relationship between m6Ascore and the biological process of SARC, for which we conducted GSEA. GSEA using TCGA data of the hallmark gene sets indicated that in the two cohorts, DNA repair, E2F targets, G2M checkpoint, mitotic spindle, mTORC1 signaling, MYC targets v1, MYC targets v2, oxidative phosphorylation, protein secretion, and unfold protein response were significantly enriched in the high-risk group (the top ten pathways are shown in Figure 8A), while the angiogenesis was significantly enriched in the low-risk group (the one pathway is shown in Figure 8A; red line). This finding provided insights into the potential biological processes and signaling pathways modulated by m^6^A-related RNAs in SARC.

The expression of immune checkpoints was used to predict immunotherapeutic benefits in multiple malignancies. Next, the study was aimed to explore whether m6Ascore could predict immunotherapeutic benefits in SARC patients. We analyzed the correlation between the high- and low-m6Ascore groups and 50 immune checkpoints. The expression of CD44 and VTCN1 was increased in the high-m6Ascore group, while the expression of TMIGD2, TNFRSF18, TNFRSF25, TNFRSF4, TNFRSF8, and NRP1 was increased in the low-m6Ascore group (Figure 8B). A chord chart was used to display the correlation between m6Ascore and immune checkpoint molecules. The results showed that m6Ascore was negatively correlated with the expression of TMIGD2, TNFRSF18, TNFRSF25, TNFRSF4, TNFRSF8, and NRP1 (Figure 8C), indicating that the poor prognosis of high-m6Ascore patients might be due to the tumor immunosuppressive microenvironment. We also investigated the differences in the distribution of infiltrating immune cells between the high-risk and low-risk groups using CIBERSORT, ESTIMATE, ssGSEA, and TIMER algorithms. ESTIMATE score and immune cell types, which were differentially infiltrated between the low- and high-risk groups, are presented in Figure 8D. To analyze the composition of immune cells in different m6Ascore subgroups, we used the Wilcoxon rank-sum test to compare the distribution of immune cells in different m6Ascore subgroups. The results showed that only in CIBERSORT algorithm, the abundance of immune cells including macrophages M0 (*p* < 0.05) and T-cell CD4 memory activated (*p* < 0.05) was highly infiltrated in the high-m6Ascore subgroup, while monocytes (*p* < 0.05) and mast cells activated (*p* < 0.05) were more abundant in the low-m6Ascore subgroup (Figure 8E). Totally, these findings suggest that there are significant associations between m6Ascore and the tumor immune landscape in SARC.

### Mutation status in SARC patients in the high- and low-m6Ascore groups

**FIGURE 9:**
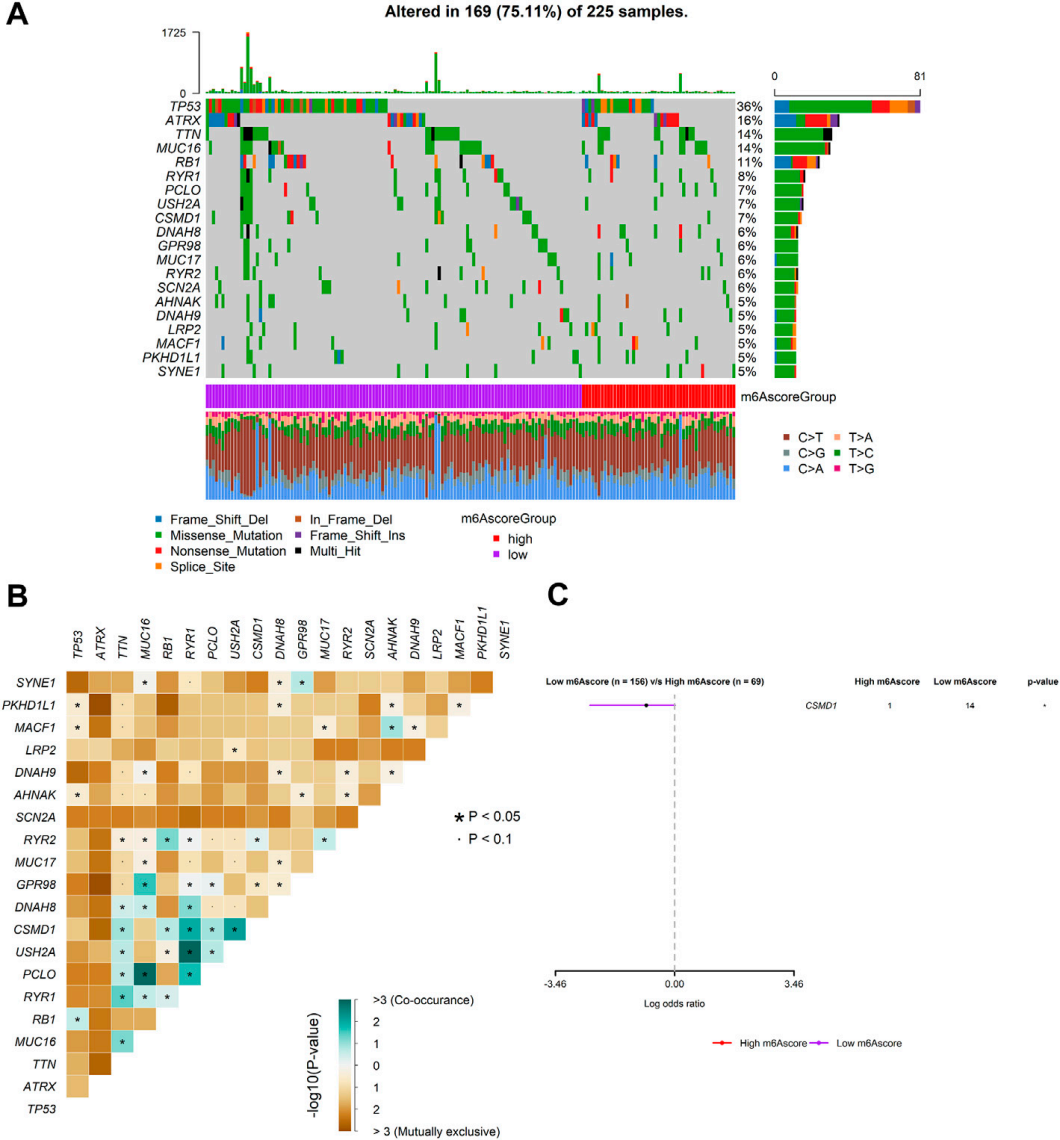
Mutation patterns of SARC patients. **(A)** Mutational patterns of 169 SARC patients in the low- and high-m6Ascore groups displayed by the oncoplot; **(B)** interaction effect of genes mutating differentially in patients in the low- and high-m6Ascore groups; and **(C)** forest plot of genes mutating differentially in patients of the low- and high-m6Ascore groups.

To investigate m6Ascore-related mechanisms in SARC, somatic mutations data were also analyzed. The frequency of mutations in top 20 genes between the two groups is shown in Figure 9A. A significant mutually exclusive phenomenon was observed among mutations of these genes (Figure 9B). Subsequently, differentially mutated genes between the two groups were detected, and the “maftools” package analysis result showed that CSMD1 was the only one significant differentially mutated gene detected between the high- and low-m6Ascore cohorts (Figure 9C). The CSMD1 mutation burden in the low-m6Ascore subtype was significantly higher than that in the high-m6Ascore subtype.

**FIGURE 10:**
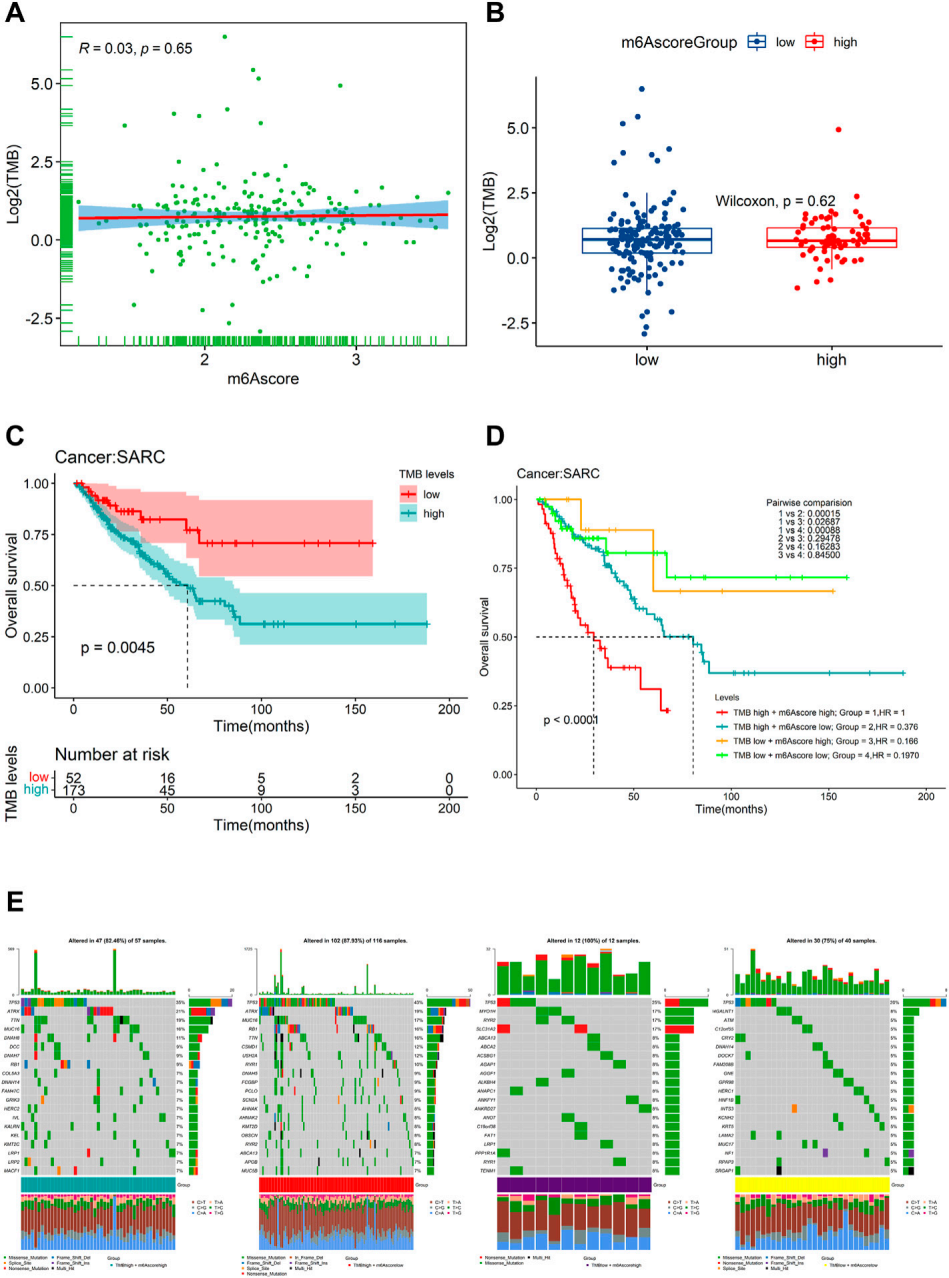
Relationship of the m6Ascore signature with TMB. **(A)** correlation between the m6Ascore signature and TMB depicted by scatter plots; **(B)** comparison of TMB between the high- and low-m6Ascore groups; **(C)** KM survival curves for the high- and low-TMB groups stratified at the optimal cutoff in TCGA-SARC cohorts (log-rank test, *p* = 0.0045); **(D)** Kaplan–Meier survival analysis for four groups stratified by combining the TMB and the m6Ascore signature in TCGA-SARC cohort; and **(E)** waterfall plot of tumor somatic mutation established by stratifying with m6Ascore and TMB. Each column represents an individual patient. Group1, high TMB /high m6Ascore, green; Group2, high TMB/low m6Ascore, red; Group3, low TMB/high m6Ascore, purple; and Group4, low TMB/low m6Ascore, yellow. The mutational types include frame shift del, frame shift ins, in-frame del, in-frame ins, missense mutation, multi-hit, nonsense mutation, and splice site.

We further found that there was no significant correlation between m6Ascore and TMB (Figure 10A). Also, there was no significant difference in TMB between the patients with high m6Ascore and those with low m6Ascore (Figure 10B). However, we found that low TMB was associated with good OS (Figure 10C, log–rank test, *p* = 0.0045). Subsequently, we explored whether the combination of m6Ascore and TMB could be a more powerful predictive biomarker for prognosis. We integrated m6Ascore and TMB to stratify all the samples into the high-TMB/low-m6Ascore, low-TMB/low-m6Ascore, high-TMB/high-m6Ascore, and low-TMB/high-m6Ascore groups. As shown in Figure 10D, significant differences were found among all groups (log-rank test, *p* < 0.0001), and patients in the high-TMB/high-m6Ascore group exhibited poor OS. These results together strongly demonstrated that the risk score was positively correlated with tumor malignancy. Next, the “maftools” R package was used to analyze and summarize the mutation data. The top 20 driver genes with the highest alteration frequency between the aforementioned subgroups are shown in Figure 10E.

### m6Ascore prediction of response to chemotherapy and immunotherapy

**FIGURE 11:**
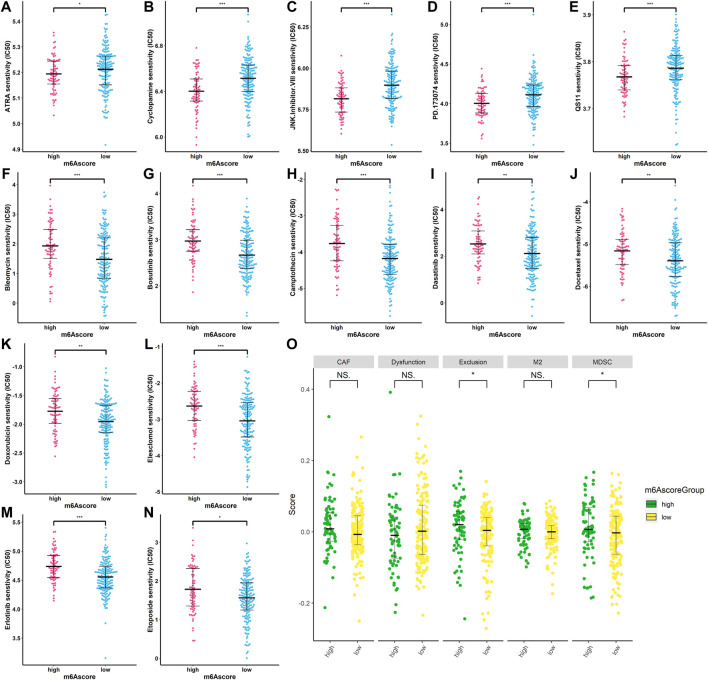
Correlation between m6Ascore and therapy. **(A–N)** IC_50_ of some chemotherapeutic drugs are in the high- and low-risk patients. *p < 0.05; **p < 0.01; ***p < 0.001; **(O)** TIDE prediction difference in the high- and low-risk patients. *p < 0.05; **p < 0.01; ***p < 0.001.

To find the potency of m6Ascore as a biomarker for predicting the response of SARC patients to drugs (including chemotherapy, targeted therapy, and immunotherapy), we used the “pRRophetic” algorithm to estimate the therapeutic response based on the half-maximal inhibitory concentration (IC_50_) available in the Genomics of Drug Sensitivity in Cancer (GDSC) database for each sample. We inferred the IC_50_ values of the 138 drugs in TCGA-SARC patients. Finally, we found that 69 compounds were screened out for significant differences in the estimated IC_50_ values between the two groups, and the high-risk group was more sensitive to these compounds including ATRA, cyclopamine, JNK inhibitor, PD173074, and QS11,while the low-risk group was more sensitive to the remaining compounds (Supplementary Figures S1–S3). Figures 11A-N display the top 14 compounds that might be used for further analysis in patients with SARC. In terms of response to immunotherapy, we used the TIDE algorithm to assess the potential clinical efficacy of immunotherapy in different m6Ascore subgroups. The TIDE algorithm assessed expression signatures of T-cell dysfunction and T-cell exclusion to assess tumor immune evasion and integrated them into the TIDE total score. In addition, the TIDE module was used to analyze multiple signatures to estimate tumor immune evasion, such as MDSC, M2 TAM, or CAF signatures. In our results (Figure 11O), the high-m6Ascore subgroup had a relatively higher T-cell exclusion score (*p* < 0.05), but there was no difference in the T-cell dysfunction scores between the two subgroups (*p* > 0.05). For other features produced by TIDE, although there was no significant difference in M2 TAM and CAF features between the two groups, the low-risk group had a relatively lower trend, and a lower proportion of MDSC was associated with the low-risk group (*p* < 0.05). These results suggest that SARC patients in the high-risk group may have a higher potential for the immunosuppressive TME status and be less responsive to ICI therapy. These findings identified the promising role of this risk signature as a predictor for chemotherapy and immunotherapy efficacy in the treatment of SARC patients.

### Construction and evaluation of the nomogram predicting OS based on the m^6^A-related signature

**FIGURE 12:**
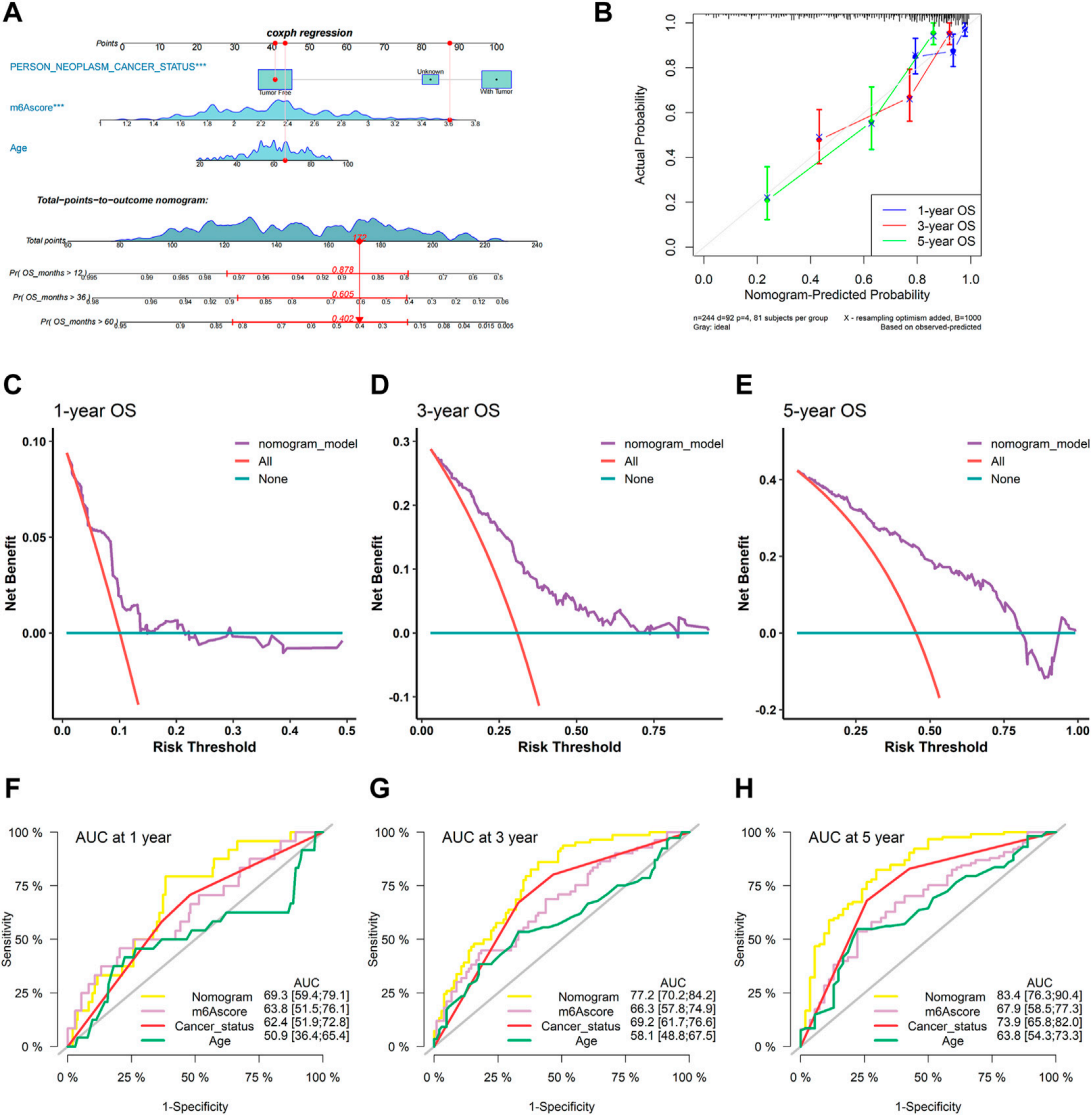
Construction and validation of the nomogram. **(A)** Nomogram based on the multivariate Cox regression model of SARC patients; **(B)** calibration plots for the internal validation of the current nomogram; the x-axis represents the nomogram-predicted overall survival, and the y-axis represents the actual overall survival of patients with SARC; **(C–E)** DCA of the nomogram based on m6Ascore for 1-, 3- and 5-year OS prediction; and **(F–H)** time-dependent ROC curves of nomogram, m6Ascore, cancer status, and age at 1, 3, and 5 years.

To confirm whether the m^6^A-related signature for OS was an independent prognostic factor, univariate and multivariate Cox regression analyses were performed (Table 3). As the results showed, in the univariate Cox regression analysis, m6Ascore, age, and cancer status were significantly associated with the OS of sarcoma patients. Then, m6Ascore, age, and cancer status were identified as independent prognostic factors of sarcomas via multivariate Cox regression analysis. All these independent factors were combined to establish a nomogram for predicting the 1-, 3-, and 5-year OS (Figure 12A). As shown in Figure 12A, m6Ascore contributes more to the total score than other variables. The 1-, 3-, and 5-year OS rates of patients declined as the total score increased. The C-index of the nomogram model reached 0.744 (95% CI: 0.707–0.784). The calibration plots showed that the nomogram model predicted the overall survival of patients with SARC well (Figure 12B). We compared the clinical net benefit of the nomogram through DCA curves. The nomogram demonstrated a larger net benefit within most of the threshold probabilities (Figures 12C–E), indicating that the nomogram had high potential clinical utility for predicting prognosis in patients with SARC. Finally, Figures 12F–H show the predictive potential of the nomogram using time-dependent ROC curves. The area under the ROC curve (AUC) of the nomogram model for OS was 0.693 at 1 year, 0.772 at 3 years, and 0.834 at 5 years.

**TABLE 3:** Univariate and multivariate Cox regression analysis of the overall survival (OS) of patients with SARC.

Variable	Univariate analysis			Multivariate analysis		
	HR	95% CI	P	HR	95% CI	P
m6Ascore	2.58	1.62–4.11	<0.0001	3.08	1.84–5.17	<0.0001
Age	1.02	1.01–1.04	0.009	1.02	1.00–1.03	0.054
Sex						
Female	1					
Male	0.79	0.52–1.19	0.263			
Cancer status						
Tumor free	1			1		
With tumor	6.07	3.54–10.43	<0.0001	6.41	3.71–11.07	<0.0001
Unknown	3.33	1.61–6.89	0.001	3.68	1.75–7.76	0.001
Race						
Asian	1					
Black or African American	1.08	0.13–8.87	0.944			
White	0.79	0.11–5.75	0.813			
Unknown	3.41	0.35–32.9	0.288			
Radiation therapy						
No	1					
Yes	0.83	0.52–1.33	0.441			
Unknown	0.38	0.05–2.76	0.341			

HR, hazard ratio; CI, confidence interval.

## Discussion

In the present study, the prognostic significance of 21 m^6^A RNA methylation regulators in SARC was first explored. The study showed that only CBLL1 was found to be an independent risk factor for OS by univariate and multivariate Cox regression analyses. Numerous studies have shown that m^6^A regulator-related signatures and m^6^A regulator-related miRNA or lncRNA signatures may serve as prognostic biomarkers in patients with various types of cancer (Li et al., 2021c; Jin et al., 2021; Xu et al., 2021). However, the expression and functional roles of m^6^A RNA methylation regulators and their associated regulatory networks in the occurrence and progression of SARC have not been widely discussed. Therefore, in this study, bioinformatics was used to analyze the existing sequencing datasets of integrated sarcoma, and miRNAs and lncRNAs related to m^6^A regulators were obtained through correlation analysis, and the upstream miRNAs and lncRNAs of m^6^A regulators were determined using miRTarBase and LncBase v.2 databases, respectively. The two parts of the results of miRNAs and lncRNAs were intersected to key miRNAs and lncRNAs, respectively. Finally, a lncRNA–miRNA–m^6^A regulator ceRNA network was constructed, which contained 104 lncRNAs, 16 miRNAs, and 11 m^6^A regulators. We should pay more attention to screening out key lncRNAs, miRNAs, and m^6^A regulators that can predict OS. Therefore, considering all these genes in the ceRNA network, we performed univariate Cox regression analysis to identify genes associated with clinical prognosis in sarcoma patients. Eight genes were found to be significantly associated with clinical outcomes in sarcoma. Finally, LASSO regression analysis and multivariate Cox regression analysis were performed, and three genes (*RP11-283I3.6*, *hsa-miR-455-3p*, and *CBLL1*) were identified as prognostic biomarkers for SARC, and the three genes were included in the risk scoring model for predicting OS for SARC.

By searching for these genes in the PubMed database, it is found that the mechanisms of *hsa-miR-455-3p* and *CBLL1* in tumor progression or studies related to tumor progression have been reported. The aberrant expression of hsa-miR-455-3p and its prognostic value have been widely reported in various types of human cancers. In our study, hsa-miR-455-3p served as a risk biomarker for SARC, consistent with the results of bioinformatics analysis by Wang et al. (2019), which showed that the differential expression level of miR-455-3p was the most significant in gliomas. Subsequently, its expression in glioma patients was examined. Consistent with the results of bioinformatics analysis, the expression level of miR-455-3p was significantly upregulated in glioma tissues compared with normal tissues. Cox regression analysis further identified miR-455-3p as an independent prognostic indicator of overall survival in glioma patients. hsa-miR-455-3p is overexpressed in skin basal cell carcinoma (BCC) (Sand et al., 2012). However, the expression of miR-455-3p is obviously decreased in the tissues and cells of hepatocellular carcinoma (HCC). This miRNA can impair HCC cell malignancy via suppression of insulin growth factor receptor expression, thereby disrupting glycolysis (Hu et al., 2019; Jiang et al., 2019). Gao et al. (2018), Yang et al. (2017), and Shang et al. (2021) reported that low expression of miR-455-3p in non-small-cell lung cancer, esophageal squamous cell carcinoma (ESCC), and pancreatic cancer (PAAD) tissues was strongly associated with poor prognosis. miR-455-3p acts as a tumor suppressor in esophageal squamous cell carcinoma (ESCC) and inhibits cell proliferation and invasion by targeting FAM83F. miR-455-3p is involved in increasing the expression of HOXC4, promoting transcriptional disorders in cancer. The miR-455-3p–HOXC4 axis is expected to be closely related to the metastasis and prognosis of human pancreatic cancer. Yi et al. (2020) and Sun et al. (2020) together showed that the expression of miR-455-3p was decreased in osteosarcoma tissues and cell lines. Patients with high miR-455-3p expression had satisfactory survival rates. miR-455-3p is a potential clinical therapeutic target and prognostic biomarker inhibiting proliferation, migration, and invasion and enhancing apoptosis. However, Hisaoka et al. (2011) showed that, compared to normal skeletal muscle, hsa-miR-455-3p was significantly upregulated in synovial sarcomas, suggesting that the molecule has a potential oncogenic role, which calls for further investigation to develop a better understanding of the oncogenic mechanisms. Our finding is consistent with this study. *CBLL1* plays an important role in tumorigenesis. Hui et al. (2019) found that *CBLL1* was upregulated in non-small-cell lung cancer (NSCLC) tissues compared to the adjacent nontumor tissues, and the high expression of *CBLL1* was associated with the tumor size in NSCLC tissues. Their results confirmed that *CBLL1* promoted the proliferation by promoting G1/S cell cycle transition in NSCLC cells. Moreover, *CBLL1* knockdown inhibited cell invasion via increased E-cadherin protein expression and decreased expression of MMP2 and MMP9 in NSCLC cell lines. Previous studies found that *CBLL1*, an E3 ubiquitin ligase, inhibits ER pathway activity by binding to an ER co-activator and then further inhibits the proliferation and differentiation of BC cells (Makdissi et al., 2009). Zheng et al. (2021) confirmed that higher *CBLL1* expression was associated with a better prognosis in BC than lower *CBLL1* expression. Functional analysis showed that *CBLL1* was related to the ESR1-related pathway, apoptosis-related pathway, cell cycle pathway and immune-related pathway in BC. Although there is no report on the correlation between the expression of RP11-283I3.6 and CBLL1 and SARC progression, based on our results, we speculate that they may be potential biomarkers for sarcoma prognosis. The mechanism of action of these genes in sarcoma is unclear and requires further follow-up studies. Subsequently, based on this risk score feature, we divided all SARC patients into the high-risk and low-risk groups, and KM survival analysis showed that patients in the low-risk group had significantly higher OS than those in the high-risk group. The time-dependent AUC indicated that the risk scoring model had good predictive performance for OS.

The molecular heterogeneity features between high- and low-risk patients were further analyzed. GSEA showed that DNA repair, E2F targets, G2M checkpoint, mitotic spindle, mTORC1 signaling, MYC targets v1, MYC targets v2, oxidative phosphorylation, protein secretion, and unfold protein response were significantly enriched in the high-risk specimens. Meanwhile, activation of angiogenesis was detected in low-risk specimens. The tumor immune microenvironment comprising stromal cells and immune cells correlates with immunotherapy response (Zhou et al., 2020). Components of the immune microenvironment are key determinants of prognosis and response to immunotherapy (Das et al., 2020). Immunotherapy is an emerging new approach to treating a variety of cancers, including sarcomas. Exploring which patient can benefit from immunotherapy remains a great challenge. Here, the association between immune cell infiltration and this risk score was comprehensively analyzed using the CIBERSORT, ESTIMATE, ssGSEA, and TIMER algorithms in the present study. Compared with patients in the high-risk group, the low-risk patients had higher levels of monocytes and mast cells activated and decreased levels of macrophages M0 and T-cell CD4 memory activated infiltration. The infiltration of M0 macrophages is positively related to poor clinical outcomes in human malignancies, including STS, which is in accordance with the findings of our study (Zhu and Hou, 2020). Thus, the worse clinical outcomes of the high-risk group may be associated with infiltrating immune cellular populations. In addition, expression levels of several immune checkpoints, including TMIGD2, TNFRSF18, TNFRSF25, TNFRSF4, TNFRSF8, and NRP1, were also higher in the low-risk group. These data were indicative of this risk signature being closely related to immunotherapy. m6Ascore may be used as an indicator independent of TMB expression to predict the efficacy of ICB. Tumor mutational burden (TMB) has been identified as a biomarker of immunotherapy response (Hellmann et al., 2018a; Hellmann et al., 2018b), where higher TMB predicts higher benefits from immunotherapy (Hellmann et al., 2018a). However, the prognostic value of TMB varies across cancer types according to a pan-cancer study (Ding et al., 2018). In bladder urothelial carcinoma (BLCA), stomach adenocarcinoma (STAD), and uterine corpus endometrial carcinoma (UCEC), high TMB is associated with longer overall survival (OS). In HNSCC, kidney renal clear cell carcinoma (KIRC), and low-grade glioma (LGG), high TMB is associated with shorter OS. The Cox regression result of TMB in SARC patients showed that HR (95% CI) was 1.25 (0.63–2.49). In the present study, patients with lower TMB had better prognosis than SARC patients with higher TMB, which is consistent with the trend of pan-cancer research. The significance of the results may be that the selected threshold is different. However, we did not observe a significant correlation between m6Ascore and TMB, nor did we observe a significant difference in the distribution of TMB between patients in the two different risk groups. The main reason is that with a threshold of *p* < 0.05, using Fisher’s exact test, *CSMD1* was the only one significant differentially mutated gene detected between the high- and low-m6Ascore cohorts, and *CSMD1* was found to be mutating more in the low-m6Ascore group, which resulted in a similar gene mutation status in the two groups. These findings suggested that TMB and m6Ascore are independent biomarkers/indicators for predicting ICB response. Interestingly, the combination of TMB and the risk characteristic we constructed can more clearly and accurately stratify SARC patients. Compared with other subgroups, patients with high TMB/high m6Ascore have the worst prognosis, which also shows that the TMB status does not affect the prognostic value of m6Ascore. m6Ascore has the potential to predict immunotherapy responsiveness, which may be independent of TMB (Zhang et al., 2020; Guan et al., 2021; Lin et al., 2021; Sun et al., 2021; Wu et al., 2021). In addition, the TIDE results suggested that the patients in the low-risk group may respond better to immunotherapy. Based on estimated IC_50_ values, the patients in the low-risk group showed sensitive chemotherapy responses to most drugs. Taken together, these findings suggest that this risk signature may play a role in the ICB treatment of SARC. Subsequent studies further confirmed that risk characteristic is independent prognostic factors in patients with sarcoma. Based on risk score and other clinically independent predictors, a nomogram for personalized clinical outcome prediction was established in the study, which was certified to perform well for predicting the 1-, 3-, and 5-year survival rates of SARC patients, showing a C-index of 0.744 (95% CI: 0.707–0.784).

Although potential biomarkers involved in tumorigenesis in a large number of samples were identified by the bioinformatics approach, it should be noted that this study also has some limitations as follows: 1) due to the lack of RNA-seq or microarray data in SARC patients, only TCGA data were included. Also, the SARC samples lacked some additional clinical follow-up information; therefore, factors such as the presence of other health conditions in patients to differentiate prognostic biomarkers were not included. 2) We internally verified the nomogram prediction model based on m6Ascore, and the findings of this study would be more meaningful if this model could be well validated externally with another real-world, independent, large-quantity, and high-quality cohort, and thus, a more diverse patient population could be extrapolated. However, the application of the prognostic prediction model based on m6Ascore required four types of data, namely, clinical data, RNA-seq (mRNAs and lncRNAs), and miRNA-seq, which involves high costs and is not easily feasible in practice. 3) Most importantly, experimental validation is needed to confirm these results and further explore the potential mechanism and role of these potential biomarkers in SARC. However, our findings showed that the nomogram model based on m6Ascore may be promising for clinical prediction of prognosis and might contain potential biomarkers for treatment response prediction for SARC patients, which remains an instructive and efficient way for predicting the accurate individual clinical outcomes of SARC patients.

## Conclusion

In conclusion, in our study, a ceRNA network based on m^6^A-related genes was successfully constructed through bioinformatics analysis of TCGA database. Candidate biomarkers in the ceRNA network were used to establish a risk profile of m^6^A-related RNAs, which is significantly associated with the prognosis and immune microenvironment of SARC, and could effectively predict the prognosis and treatment efficacy of STSs. The results of this study suggest that these markers may play an important role in the therapeutic target and prognostic analysis of sarcoma patients.

## Data Availability

The original contributions presented in the study are included in the article/Supplementary Material, further inquiries can be directed to the corresponding author.
