# Supplementary figures and images for: The development of a novel signature based on the m6A RNA methylation regulator-related ceRNA network to predict prognosis and therapy response in sarcomas
Source: Front Genet. 2022 Oct 12;13:894080. doi: 10.3389/fgene.2022.894080 (PMC9597465; doi:10.3389/fgene.2022.894080)

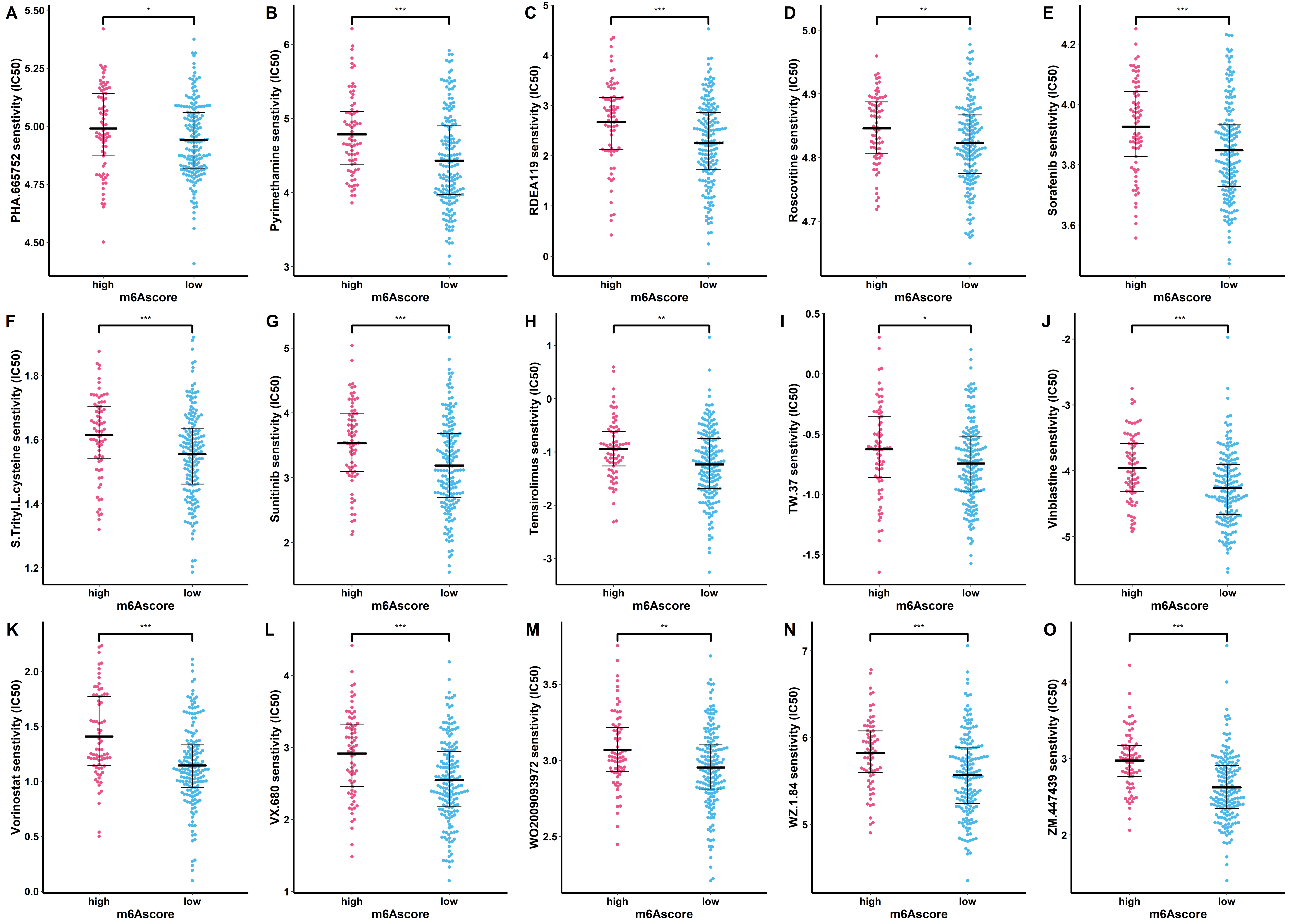

Supplement: Supplementary file 2 [file DataSheet2.ZIP › Supplementary Image/Supplementary Figure S3.tiff]
